# Correlations between Dysphagia Severity Scale Scores and Clinical Indices in Individuals with Multiple System Atrophy

**DOI:** 10.1002/mdc3.70055

**Published:** 2025-03-25

**Authors:** Ryunosuke Nagao, Yasuaki Mizutani, Kazuya Kawabata, Junichiro Yoshimoto, Yoko Inamoto, Seiko Shibata, Mizuki Ito, Yohei Otaka, Hirohisa Watanabe

**Affiliations:** ^1^ Department of Neurology Fujita Health University School of Medicine Toyoake Aichi Japan; ^2^ Division of Brain Thera Informatics, International Center for Brain Science Fujita Health University Toyoake Aichi Japan; ^3^ International Center for Brain Science Fujita Health University Toyoake Japan; ^4^ Department of Biomedical Data Science Fujita Health University School of Medicine Toyoake Aichi Japan; ^5^ Faculty of Rehabilitation, School of Health Sciences Fujita Health University Toyoake Aichi Japan; ^6^ Department of Rehabilitation Medicine, School of Medicine Fujita Health University Toyoake Aichi Japan

**Keywords:** multiple system atrophy, dysphagia severity scale, 5‐hydroxyindoleacetic acid, serotonergic neurons, disease severity

## Abstract

**Background:**

Dysphagia significantly impacts prognosis in individuals with multiple system atrophy (MSA). While video‐based assessments are practical, their limited availability highlights the need for a simple tool such as the Dysphagia Severity Scale (DSS) in clinical practice.

**Objectives:**

To evaluate the utility of the DSS in assessing dysphagia in MSA patients and its correlations with clinical indices.

**Methods:**

We examined 43 MSA patients using the DSS and other clinical measures, including the Unified MSA Rating Scale (UMSARS) and cerebrospinal fluid 5‐hydroxyindoleacetic acid levels. As a follow‐up, 11 of 43 patients underwent a secondary DSS evaluation. Spearman's correlation and linear mixed models were used to analyze cross‐sectional and longitudinal relationships.

**Results:**

DSS scores were significantly correlated with UMSARS Parts 1, 2, and 4, as well as disease duration and blood pressure changes. This indicates that the DSS is sensitive to MSA‐related motor and autonomic dysfunctions, and that the DSS could provide a more detailed assessment of swallowing function compared with the UMSARS Part 1 swallowing subscore. Additionally, DSS score was correlated with cerebrospinal fluid 5‐hydroxyindoleacetic acid levels. Our longitudinal analysis further supported the role of DSS score as a reliable marker of dysphagia progression over time.

**Conclusions:**

The DSS is a sensitive and practical tool for evaluating dysphagia. Thus, combining the DSS and UMSARS could improve dysphagia monitoring in individuals with MSA. Our data support the use of the DSS as a valuable clinical and research tool in MSA management.

Multiple system atrophy (MSA) is a sporadic, adult‐onset neurodegenerative disorder that can be classified as predominant parkinsonism (MSA‐P) or cerebellar ataxia (MSA‐C).^1^ It is pathologically defined according to the presence of α‐synuclein inclusions in oligodendroglia.^2^ The Movement Disorder Society criteria for diagnosing MSA (MDS‐MSA) include “severe dysphagia within 3 years of motor onset” as one of the supportive clinical features.^1^ Swallowing impairments in MSA patients can affect the oral preparation, oral, pharyngeal, and esophageal^3^ phases of swallowing, leading to delayed bolus transport, insufficient tongue movement, and retention in the oral phase, as well as slow laryngeal elevation, residue, and pharyngeal constriction in the pharyngeal phase.^4^, ^5^, ^6^, ^7^, ^8^

The Neuromuscular Disease Swallowing Status Scale, associated with the functional oral intake scale, assesses dysphagia severity in MSA patients^9^ and is correlated with pneumonia risk and poorer prognosis.^10^, ^11^ While videofluoroscopy (VF) and videoendoscopy (VE) assessments can detect asymptomatic dysphagia,^4^, ^5^, ^6^ these tools are not widely accessible. Thus, a reliable, easily applicable scale is needed. Although the Unified MSA Rating Scale (UMSARS) Part 1 was designed to be patient‐centered,^12^ its swallowing subscore lacks sensitivity to changes and does not account for food consistency.^13^ In contrast, the Dysphagia Severity Scale (DSS), developed in 1999 by Saitoh and colleagues as part of a Japanese Ministry of Health and Welfare project,^14^ has gained widespread acceptance in Japan for both clinical and research purposes because of its simplicity and practicality in bedside swallowing evaluations.^15^, ^16^ Over the past decade, the DSS has also achieved international recognition. This is evidenced by its inclusion in an English‐language dysphagia textbook^17^ and its utilization in international studies on dysphagia risk, nutrition, and functional outcomes.^18^, ^19^, ^20^ Therefore, the DSS combines ease of use with high inter‐rater reliability, providing a practical alternative for dysphagia assessment in MSA patients, particularly in settings without advanced imaging.^21^, ^22^, ^23^, ^24^ However, the relationship between DSS score and the broader clinical severity of MSA has not yet been examined.

A notable feature of MSA is the high incidence of sudden death, with severe autonomic dysfunction being a major risk factor. Notably, patients who experience sudden death or exhibit severe autonomic dysfunction demonstrate a significant reduction in serotonergic neurons in the brainstem.^25^, ^26^, ^27^ We previously demonstrated that levels of CSF 5‐HIAA, the major serotonin metabolite, were significantly lower in patients with MSA than in controls. Moreover, CSF 5‐HIAA levels were inversely correlated with UMSARS total scores, including the UMSARS Part 1 swallowing subscore, suggesting a potential role of serotonin in motor dysfunction in MSA patients.^28^

This study aimed to clarify the role of the DSS in dysphagia assessment and its association with clinical indices in MSA. Understanding these relationships could support the integration of the DSS into routine MSA monitoring, which could ultimately enhance dysphagia management and improve patient outcomes.

## Methods

### Participants

We included 43 patients with MSA who had been admitted to our hospital between January 2020 and August 2024. Patients underwent swallowing assessments and clinical score evaluation and were diagnosed with clinically established MSA according to the MDS‐MSA criteria.^1^ All assessments were conducted in the absence of serotonergic drugs. MSA‐P and MSA‐C were mainly distinguished according to dominant symptoms, as evaluated by the physician in charge of the examination. Of the 43 patients, 19 had MSA‐P and 24 had MSA‐C. There were 18 men and 25 women, with a mean age at examination of 63.40 ± 9.34 years and a mean disease duration of 33.42 ± 21.45 months. Levodopa was administered to 25 patients and the total levodopa equivalent daily dose (LEDD) was calculated for each participant according to the established formula.^29^

### Clinical Assessments

This study had both cross‐sectional and longitudinal components. In the cross‐sectional part, we collected DSS scores, CSF 5‐HIAA levels, and assessed clinical severity using the UMSARS in 43 MSA patients. We also evaluated changes in systolic blood pressure (sBP), diastolic blood pressure (dBP), heart rate (HR), and HR/sBP in UMSARS Part 3. We analyzed the correlations between DSS scores, UMSARS scores, and CSF 5‐HIAA levels. Next, we compared DSS scores and clinical indices between the MSA‐P and MSA‐C groups.

In the longitudinal component, we analyzed data from 11 of the 43 MSA patients who underwent secondary evaluations. In this group, DSS and clinical scores were collected for a mean period of 21.36 ± 12.09 months following the initial evaluation. In a considerable number of the patients, follow‐up clinical and swallowing evaluations were inadequate and were not conducted at the same time. As a result, data from only 11 cases were available for longitudinal evaluation.

### DSS

The DSS is a seven‐level classification system that can easily be implemented in daily clinical practice (Table 1).^21^, ^22^, ^23^, ^24^ Visual evaluations such as VE or VF are not always necessary for determining DSS level and this can be clinically assessed when such tests are not available.

**TABLE 1:** Dysphagia severity scale

7: Within normal limits	No clinical problems.
6: Minimal problems	Mild problems including subjective complaints.
5: Oral problems	Significant problems in the oral preparatory or oral phase without aspiration.
4: Occasional aspiration	Occasional aspiration or clinically suspected aspiration with marked pharyngeal residue.
3: Water aspiration	Aspiration with thin liquid but no aspiration with adjusted food.
2: Food aspiration	Aspiration of everything with no effect of compensatory technique, but stable respiratory condition.
1: Saliva aspiration	Aspiration of everything including saliva or no swallowing reflex and unstable medical condition.

In a previous report, DSS scores evaluated by a certified dysphagia nurse had a 91% agreement rate with DSS scores evaluated via endoscopic swallowing testing. This finding suggests that the swallowing assessment scale exhibits high inter‐rater reliability.^30^ In the present study, a speech therapist determined the severity of swallowing dysfunction based on history (reports of eating patterns and swallowing ability), dietary observation, a repeated salivary swallowing test, and a water swallowing test.

### CSF 5‐HIAA Levels

CSF 5‐HIAA levels were measured via high‐performance liquid chromatography according to previously described methods.^31^ CSF was stored at 4°C after collection and analysis was conducted within 1 day. CSF 5‐HIAA level was considered unchanged during the storage period based on a previous study.^32^ The molecular weight of 5‐HIAA was calculated to be 191 g/mol and the measurement unit was standardized.

### Statistical Analysis

Continuous variables are expressed as the mean ± SD. Differences were considered statistically significant at *p* < 0.05. Fisher's exact test was used to compare sex distributions. The normality of the variables was validated using the Shapiro–Wilk test. The Wilcoxon rank‐sum test was used to compare the continuous variables between the two groups. Correlations between continuous variables, including DSS score and clinical indices, were assessed using the Spearman's rank correlation coefficient. We applied false discovery rate (FDR) correction using the Benjamini–Hochberg procedure for multiple comparisons between DSS scores and UMSARS Part 1 and Part 2 subscores.^33^

Linear mixed models were used in the longitudinal analysis to assess the relationship between DSS scores and various clinical indices in MSA patients. Both log‐transformed DSS (log DSS) and non‐log‐transformed DSS (non‐log DSS) scores were analyzed to evaluate their association with disease progression, allowing for the detection of incremental changes in dysphagia over time. Logarithmic transformation of DSS scores reduced the sample error and improved the accuracy of the model given the small number of cases, variability of the distribution, and low score values. The primary outcome variables were DSS scores and log DSS scores. The main predictor variables included evaluation interval, disease duration, swallowing items from UMSARS Part 1, and total UMSARS score (UMSARS Part 1 + 2 + 4). For each model (log DSS and non‐log DSS), the longitudinal data were analyzed using mixed models with a random intercept for patient ID to account for repeated measurements within subjects—the random effect of patient ID controlled for inter‐individual variability in baseline dysphagia severity. The fixed effects included disease duration, UMSARS scores, and evaluation interval, which enabled us to investigate how these factors influenced dysphagia progression. We calculated Akaike Information Criterion (AIC) values to compare the fit of the log DSS and non‐log DSS models. Lower AIC values indicated a better model fit, and this criterion was used to determine the model that best captured the variability in dysphagia progression.

The *p*‐values for the fixed effects were used to determine the statistical significance of the associations between DSS scores and clinical variables. Statistical significance was set at *p* < 0.05. For the first and second evaluations, we assessed the cross‐sectional correlations between DSS scores and clinical indices using Spearman's rank correlation coefficients. Longitudinal changes (Δ) in DSS scores were compared with changes in the other clinical scores, although the correlations for longitudinal changes were weaker because of individual variability in disease progression. The objective of these analyses was to accommodate inter‐individual differences among patients and to ascertain the strength of the correlation between DSS and UMSARS scores, as well as the swallowing items from UMSARS Part 1.

All statistical analyses were conducted using JMP Pro 16.0 software (SAS Institute Inc., Cary, NC, USA).

## Results

### Clinical Characteristics

The clinical characteristics of the MSA‐P and the MSA‐C groups are shown in Table 2. No significant differences were observed in sex, age at onset, age at examination, or disease duration. The percentage of patients treated with dopamine (*p* = 0.035) and total LEDD (*p* = 0.0033) were both higher in the MSA‐P group. CSF 5‐HIAA levels were significantly lower in the MSA‐P group (*p* = 0.0339). However, there were no significant differences between the two groups in terms of disease severity, blood pressure fluctuations, or pulse rate changes according to UMSARS scores. Similarly, no significant differences were observed in the total scores or swallowing subscore of UMSARS Part 1 (Table 2).

**TABLE 2:** Clinical characteristics and statistical comparison of the MSA‐P and MSA‐C groups

Characteristics	MSA‐P	MSA‐C	P‐value
N	19	24	
Male / Female	9/10	9/15	0.5496
Age at onset (years)	60.68 ± 9.86	60.04 ± 9.65	0.7504
Age at examination (years)	64.05 ± 9.34	62.88 ± 9.50	0.6155
Disease duration (months)	28.21 ± 13.35	37.54 ± 25.70	0.3587
Dopaminergic treatment	16	9	**0.0035**
LEDD (mg)	305.26 ± 209.43	118.75 ± 172.46	**0.0033**
CSF 5‐HIAA (ng/dL)	9.18 ± 5.25	12.86 ± 5.40	**0.0339**
DSS score	5.53 ± 1.31	5.42 ± 1.74	0.7903
UMSARS Part I score	18.95 ± 10.76	18.92 ± 12.69	0.8929
UMSARS Part I (2): swallowing	0.82 ± 0.83	1.04 ± 1.08	0.6786
UMSARS Part II score	22.95 ± 9.20	20.58 ± 11.87	0.3520
UMSARS Part III Δ sBP (mmHg)	39.78 ± 16.37	36.36 ± 15.99	0.6049
UMSARS Part III Δ dBP (mmHg)	20.39 ± 12.06	17.41 ± 11.39	0.3540
UMSARS Part III Δ HR (bpm)	7.11 ± 5.70	9.05 ± 6.43	0.2203
Δ HR / Δ sBP	0.18 ± 0.16	0.25 ± 0.53	0.1919
UMSARS Part IV score	2.63 ± 1.30	2.42 ± 1.44	0.4582

Abbreviations: CSF, cerebrospinal fluid; dBP; diastolic blood pressure; DSS, dysphagia severity scale; HR, heart rate; LEDD, levodopa equivalent daily dose; MSA, multiple system atrophy; MSA‐C, MSA‐cerebellar type; MSA‐P, MSA‐parkinsonian type; sBP, systolic blood pressure; UMSARS, unified MSA rating scale; 5‐HIAA, 5‐hydroxyindoleacetic acid.

### Correlation between DSS and UMSARS Scores

DSS scores were significantly negatively correlated with composite scores in UMSARS Part 1 (rs = −0.7279, *p* < .0001), Part 2 (rs = −0.6724, *p* = < .0001), and Part 4 (rs = −0.7519, *p* = < .0001), along with changes in sBP (rs = −0.5695, *p* = 0.0001), dBP (rs = −0.4054, *p* = 0.0095), and HR/sBP (rs = 0.4160, *p* = 0.0076) in UMSARS Part 3 (Table 3).

**TABLE 3:** Correlations between DSS scores and clinical indices as assessed using the Spearman's correlation coefficient

Clinical parameters	Mean ± SD	rs	*p*‐value
DSS score	5.47 ± 1.55		
CSF 5‐HIAA (ng/mL)	11.20 ± 5.58	0.3546	**0.0248**
Onset age (years)	60.33 ± 9.63	0.1871	0.2296
Age at examination (years)	63.40 ± 9.34	0.115	0.9416
Disease duration (months)	33.42 ± 21.45	−0.5958	<.**0001**
UMSARS Part I score	18.93 ± 11.74	−0.7279	<.**0001**
UMSARS Part II score	21.63 ± 10.71	−0.6724	<.**0001**
UMSARS Part III Δ sBP (mmHg)	37.90 ± 16.05	−0.5695	**0.0001**
UMSARS Part III Δ dBP (mmHg)	18.75 ± 11.64	−0.4054	**0.0095**
UMSARS Part III Δ HR (bpm)	8.18 ± 6.11	0.2066	0.2009
Δ HR / Δ sBP	0.22 ± 0.40	0.4160	**0.0076**
UMSARS Part IV score	2.51 ± 1.37	−0.7519	<.**0001**

Abbreviations: CSF, cerebrospinal fluid; dBP; diastolic blood pressure; DSS, dysphagia severity scale; HR, heart rate; MSA, multiple system atrophy; sBP, systolic blood pressure; UMSARS, unified MSA rating scale; 5‐HIAA, 5‐hydroxyindoleacetic acid.

### Comparison between DSS Scores and Clinical Indices

DSS scores were significantly negatively associated with disease duration (rs = −0.5958, *p* = < .0001) but not with onset age (rs = 0.1871, *p* = 0.2296) or age at examination (rs = 0.1150, *p* = 0.9416) (Table 3). Disease duration was more strongly associated with DSS scores (rs = −0.5958, *p* = < .0001) than swallowing scores from UMSARS Part 1 (rs = 0.4223, *p* = 0.0048).

### Correlation between DSS Scores, UMSARS Subscores, and CSF 5‐HIAA Levels

DSS scores were significantly correlated with the following subscores from UMSARS Part 1 and 2: Items 1, 2, 3, 4, 5, 6, 7, 8, 9, 10, 11 in Part 1 and Items 1, 2, 4, 5, 7, 8, 9, 10, 11, 12, 13, and 14 in Part 2. These items also had FDR‐adjusted *p*‐values <0.05 (Table 4). A positive correlation was observed between DSS scores and CSF 5‐HIAA levels (rs = 0.3546, *p* = 0.0248) (Table 5), however, the ordinal logistic regression analysis, which used DSS as the dependent variable, identified disease duration (*p* = 0.0021) and UMSARS Part 4 (*p* = 0.0021) scores as the primary determinants (Table 4).

**TABLE 4:** Correlations between DSS score and clinical indices as assessed using the Spearman rank correlation coefficient and FDR correction

		Mean ± SD	rs	*p*‐value	FDR (q‐value)
UMSARS Part 1					
1	Speech	1.37 ± 0.87	−0.331	0.0301	0.0341
2	Swallowing	0.95 ± 0.97	−0.646	<.0001	<.0001
3	Handwriting	1.51 ± 1.14	−0.493	0.0008	0.0011
4	Cutting food and handling utensils	1.37 ± 1.11	−0.698	<.0001	<.0001
5	Dressing	1.41 ± 1.31	−0.624	<.0001	<.0001
6	Hygiene	1.49 ± 1.28	−0.590	<.0001	0.0001
7	Walking	2.14 ± 1.23	−0.735	<.0001	0.0008
8	Falling	1.47 ± 1.45	−0.524	0.0003	0.0005
9	Orthostatic symptoms	1.47 ± 1.40	−0.640	<.0001	<.0001
10	Urinary function	1.65 ± 1.30	−0.713	<.0001	<.0001
11	Sexual function	2.51 ± 1.81	−0.253	0.1011	0.1051
12	Bowel function	1.81 ± 1.05	−0.539	0.0002	0.0004
UMSARS Part 2					
1	Facial expression	1.56 ± 1.10	−0.637	<.0001	<.0001
2	Speech	1.47 ± 0.88	−0.334	0.0287	0.0340
3	Ocular motor dysfunction	1.40 ± 0.76	−0.097	0.5353	0.5353
4	Tremor at rest	0.49 ± 0.88	−0.597	<.0001	0.0001
5	Action tremor	1.26 ± 1.03	−0.566	<.0001	0.0002
6	Increased tone	1.14 ± 0.97	−0.274	0.0750	0.0812
7	Rapid alternating movements of hands	1.79 ± 0.89	−0.449	0.0025	0.0033
8	Finger taps	1.74 ± 0.85	−0.440	0.0032	0.0040
9	Leg agility	1.70 ± 0.86	−0.531	0.0002	0.0004
10	Heel–knee‐shin test	1.74 ± 0.88	−0.490	0.0009	0.0012
11	Arising from chair	1.63 ± 1.61	−0.637	<.0001	<.0001
12	Posture	1.28 ± 1.22	−0.559	<.0001	0.0002
13	Body sway	2.12 ± 1.24	−0.538	0.0002	0.0003
14	Gait	2.23 ± 1.17	−0.684	<.0001	<.0001

Abbreviations: DSS, dysphagia severity scale; FDR, false discovery rate; MSA, multiple system atrophy; UMSARS, unified MSA rating scale.

**TABLE 5:** The results of the ordinal logistic regression analysis with DSS as the objective variable

Item	Estimate	Standard Error	Chi‐Square	*p*‐value	FDR (q‐value)
UMSARS 4	2.372	0.769	9.50	**0.0021**	**0.0021**
Disease duration (months)	0.091	0.030	9.46	**0.0021**	**0.0019**
UMSARS 2	−0.181	0.111	2.66	0.1028	0.0841
ΔHR/ΔsBP	−2.253	1.599	1.99	0.1588	0.1155
CSF 5‐HIAA	−0.129	0.105	1.52	0.2182	0.1389
ΔdBP	0.062	0.054	1.32	0.2508	0.1368
ΔsBP	0.039	0.040	0.96	0.3282	0.1492
Age at onset	0.105	0.138	0.57	0.4495	0.1635
Age	−0.070	0.136	0.26	0.6082	0.1659
UMSARS 1	0.014	0.098	0.02	0.8883	0.1615
ΔHR	−0.004	0.125	0.00	0.9775	0.0889

Abbreviations: CSF, cerebrospinal fluid; dBP; diastolic blood pressure; DSS, dysphagia severity scale; HR, heart rate; MSA, multiple system atrophy; sBP, systolic blood pressure; UMSARS, unified MSA rating scale; 5‐HIAA, 5‐hydroxyindoleacetic acid.

### Comparison of DSS Scores and Clinical Indices between the MSA‐P and MSA‐C Groups

In the MSA‐P group, DSS scores were significantly negatively correlated with UMSARS Part 1 (rs = −0.7537, *p* = 0.0002), Part 2 (rs = −0.6070, *p* = 0.0059) and Part 4 (rs = −0.7751, *p* < .0001), and positively correlated with CSF 5‐HIAA levels (rs = 0.5053, *p* = 0.0324) (Table 6). In the MSA‐C group, DSS scores were strongly negatively correlated with disease duration (rs = −0.8133, *p* < .0001), UMSARS Part 1 (rs = −0.7207, *p* < .0001) and Part 2 (rs = −0.7131, *p* < .0001), and ΔsBP in UMSARS Part 3 (rs = −0.7418, *p* < .0001) and Part 4 (rs = −0.7437, *p* < .0001) (Table 6). Additionally, DSS scores were significantly positively correlated with ΔHR/ΔsBP (rs = 0.4681, *p* = 0.0280). A comparison of DSS and UMSARS subscores is provided in Tables S1 and S2.

**TABLE 6:** The associations between DSS and clinical indices using Spearman correlation coefficient in MSA‐P and MSA‐C patients

Characteristics	MSA‐P			MSA‐C		
	Mean ± SD	rs	*p*‐value	Mean ± SD	rs	*p*‐value
DSS	5.53 ± 1.31			5.42 ± 1.74		
CSF 5‐HIAA (ng/dL)	9.18 ± 5.25	0.5053	**0.0324**	12.86 ± 5.40	0.2449	0.2721
Age at onset (years)	60.68 ± 9.86	0.3572	0.1333	60.04 ± 9.65	0.0445	0.8363
Age at examination (years)	64.05 ± 9.34	0.1464	0.5498	62.88 ± 9.50	−0.0945	0.6604
Disease duration (months)	28.21 ± 13.35	−0.1559	0.5239	37.54 ± 25.70	−0.8133	**<.0001**
UMSARS Part I	18.95 ± 10.76	−0.7537	**0.0002**	18.92 ± 12.69	−0.7207	**<.0001**
UMSARS Part II	22.95 ± 9.20	−0.6070	**0.0059**	20.58 ± 11.87	−0.7131	**<.0001**
UMSARS Part III Δ sBP (mmHg)	39.78 ± 16.37	−0.2914	0.2407	36.36 ± 15.99	−0.7418	**<.0001**
UMSARS Part III Δ dBP (mmHg)	20.39 ± 12.06	−0.4658	0.0514	17.41 ± 11.39	−0.3495	0.1108
UMSARS Part III Δ HR (bpm)	7.11 ± 5.70	0.2222	0.3755	9.05 ± 6.43	0.2327	0.2973
ΔHR/ΔsBP	0.18 ± 0.16	0.3378	0.1704	0.25 ± 0.53	0.4681	**0.0280**
UMSARS Part IV	2.63 ± 1.30	−0.7751	**<.0001**	2.42 ± 1.44	−0.7437	**<.0001**

Abbreviations: CSF, cerebrospinal fluid; dBP; diastolic blood pressure; DSS, dysphagia severity scale; HR, heart rate; LEDD, levodopa equivalent daily dose; MSA, multiple system atrophy; MSA‐C, MSA‐cerebellar type; MSA‐P, MSA‐parkinsonian type; sBP, systolic blood pressure; UMSARS, unified MSA rating scale; 5‐HIAA, 5‐hydroxyindoleacetic acid.

### Longitudinal Changes in DSS and UMSARS Scores

Table 7 shows the longitudinal changes in the clinical characteristics of 11 patients with MSA. The linear mixed model analysis was conducted with random effects in the evaluation interval. The log DSS model showed a better fit, with an AIC of 19.53 compared with 55.02 for the non‐log DSS model, indicating that it more accurately captured the variability in dysphagia progression. Disease duration significantly affected non‐log DSS scores (*p* < .0001), while the evaluation interval markedly influenced log DSS scores (*p* = 0.0430). This indicates that the timing of the assessment played a key role in detecting the progression of dysphagia. Furthermore, the data indicate that the log DSS score is particularly sensitive to small, incremental changes in swallowing function, which may be more noticeable with more frequent evaluations (Table 8).

**TABLE 7:** Longitudinal changes in clinical characteristics

Characteristics	The first measurement	The follow‐up measurement	Δ	Annual change
Disease duration, mean ± SD (range, months)	33.18 ± 18.47 (10–70)	54.55 ± 22.49 (26–86)	21.36 ± 12.09 (9–46)	
DSS score, mean ± SD (range), DSS grade (patient number)	5.91 ± 1.30 (3–7) 3(4), 5(1), 6(1), 7(5)	3.91 ± 1.70 (1–6) 1(1), 2(2), 3(1), 4(2), 5(3), 6(2)	−2.00 ± 1.34 (−5 to −1) −5(1), −4(1), −2(4), −1(5)	−1.13 ± 0.39 (−0.42 to −0.08)
UMSARS Part 1 score, mean ± SD (range)	16.91 ± 10.91 (3–41)	28.27 ± 12.12 (13–47)	11.36 ± 9.39 (2–34)	6.67 ± 4.48 (0.17–2.83)
Part 1 (2): swallowing, mean ± SD (range)	1.0 ± 0.77 (0–2)	1.82 ± 1.25 (0–4)	0.82 ± 0.98 (0–3)	0.48 ± 0.50 (0–0.25)
UMSARS Part 2 score, mean ± SD (range)	20.36 ± 10.55 (8–45)	33.73 ± 12.87 (12–53)	13.36 ± 8.29 (4–30)	8.46 ± 5.66 (0.33–2.5)
UMSARS Part 4 score, mean ± SD (range)	2.18 ± 1.25 (1–5)	3.45 ± 1.37 (1–5)	1.27 ± 1.19 (0–4)	0.85 ± 0.83 (0–0.33)

Abbreviations: DSS, dysphagia severity scale; MSA, multiple system atrophy; UMSARS, unified MSA rating scale.

**TABLE 8:** Linear mixed model analysis of the log DSS and non‐log DSS models

Model	Variable	Estimate	Standard Error	*t*‐value	*p*‐value
Log DSS Model	Intercept	2.0584	0.1016	20.26	**<.0001**
	Disease duration	−0.0068	0.0031	−2.16	0.0769
	Part 1 (2): swallowing	0.2168	0.0939	2.31	0.0541
	Total UMSARS score	−0.0076	0.0033	−2.29	0.0588
	Evaluation interval	−0.0145	0.0063	−2.31	**0.0430**
	Residual variance	3.6168e‐5	0.0007		
	Random effect (ID)	0.0223	0.0150		0.1383
Non‐Log DSS Model	Intercept	8.0560	0.6776	11.89	**<.0001**
	Disease duration	−0.0538	0.0086	−6.23	**<.0001**
	Part 1 (2): swallowing	0.0187	0.2125	0.09	0.9308
	Total UMSARS score	−0.0096	0.0223	−0.43	0.6710
	Evaluation interval	−0.0288	0.0223	−1.30	0.2126
	Residual variance	0.2348	0.0805		
	Random effect (ID)	0.5076	0.1741		**0.0036**

Abbreviations: DSS, dysphagia severity scale; MSA, multiple system atrophy; UMSARS, unified MSA rating scale.

## Discussion

In this study, we examined the association between DSS scores and various clinical indices in MSA patients via both cross‐sectional and longitudinal analyses. Our cross‐sectional findings revealed a strong correlation between DSS scores and clinical measures, suggesting that DSS scores effectively reflect motor and autonomic dysfunction in individuals with MSA. In the longitudinal analysis, DSS scores were significantly correlated with assessment intervals (disease duration), suggesting that these scores can serve as an independent marker of disease progression over time. In particular, the log‐transformed DSS model showed improved sensitivity to subtle changes, supporting its utility for monitoring progressive dysphagia in MSA. These results indicate that the DSS is a valuable tool for assessing both short‐term changes and long‐term disease progression in patients with MSA.

### Cross‐Sectional Evaluation of DSS Score

The high correlation between the DSS and other clinical scores, such as UMSARS Part 4, which assesses motor function, highlights the close relationship between dysphagia, motor decline, and autonomic dysfunction in MSA patients. This supports the clinical utility of the DSS as a comprehensive measure of disease burden in MSA. Because it reflects both motor and swallowing impairments, the DSS serves as an efficient tool for clinicians to use when assessing overall disease severity. In clinical practice, such correlations are particularly valuable because they enable a more integrated understanding of how dysphagia fits within the broader context of disease progression. Given that swallowing dysfunction significantly impacts patients’ quality of life, nutritional status, and survival, a tool such as the DSS that provides a clear and quantifiable measure of dysphagia is crucial for timely and appropriate interventions.

### Longitudinal Evaluation of DSS Score

Although the random effects were not significant in the log DSS model and we found no consistent trend across individuals in terms of evaluation intervals, the log DSS may have affected the evaluation intervals even without random effects. In addition, the log DSS model showed a better statistical fit based on AIC, suggesting that it more accurately captured the variability in the data. In particular, the evaluation interval had a significant impact on log DSS scores (*p* < 0.0430), underscoring the importance of assessment timing in detecting subtle, progressive changes in dysphagia. The log transformation may increase the sensitivity of the model to these smaller changes, particularly in the earlier stages of the disease, where an incremental decline in swallowing function may be more difficult to detect using non‐transformed scales. Additionally, the log DSS model demonstrated heightened sensitivity to changes in patients with lower initial severity scores, reflecting a trend where individuals with mild dysphagia symptoms are more likely to progress to more severe stages over longer evaluation intervals. This finding suggests that, while DSS scores were primarily correlated with disease duration, the log‐transformed model captured incremental disease progression among milder cases, making it a valuable tool for detecting early signs of deterioration.

### Comparison of DSS Scores and UMSARS Part I Subscores for Assessing Swallowing

Compared with the swallowing subscore of UMSARS Part 1, the DSS provided a more detailed and sensitive measure of dysphagia in individuals with MSA. Although the UMSARS is widely used to assess motor function in MSA patients, its swallowing subscore has limited scope and may not fully capture dysphagia progression.^13^ The finer gradation of the DSS enables the detection of subtle yet clinically relevant changes in swallowing function that may go unnoticed with the broader UMSARS scoring categories. A previous study identified five items on the swallowing disturbance questionnaire with high specificity, including food form, food residue, and the swallowing process—items notably absent from the UMSARS.^34^ The DSS, which is explicitly designed for assessing swallowing, is superior for detecting early signs and tracking dysphagia progression. In another report, DSS evaluations conducted by a certified dysphagia nurse showed a 91% agreement with the results of endoscopic swallowing tests.^30^

Given that dysphagia progression is a critical concern in MSA because of the associated aspiration risk, a tool like the DSS, which offers specific, nuanced insights into swallowing function, is invaluable for accurately tracking patient deterioration. Future clinical assessments may benefit from combining the DSS with the UMSARS, thereby integrating overall motor assessment with focused dysphagia monitoring to achieve more nuanced insight into MSA progression.

### Association between DSS Scores and UMSARS Part I and Part II Subscores

The strong correlation between DSS scores and UMSARS Parts 1 and 2 subscores suggests that dysphagia severity is closely linked to the progression of motor and autonomic symptoms in MSA patients, and that it could be a broader marker of disease progression. Dysphagia is a poor prognostic factor in MSA patients because it likely reflects the deterioration of motor and autonomic functions.^11^, ^35^ The negative correlation between DSS and UMSARS scores, particularly for swallowing, gait, and posture, highlights how worsening motor functions are correlated with dysphagia progression, reflecting declines in overall motor control. Thus, the DSS could serve as a valuable indicator of swallowing dysfunction and be useful for monitoring autonomic and motor dysfunction progression. This relationship implies potential shared pathological mechanisms within the nervous system, including regions beyond the basal ganglia or cerebellum‐brainstem circuits traditionally implicated in MSA. The correlation between dysphagia, gait, and posture supports this, suggesting system‐wide dysfunction rather than isolated lesions. Alternatively, dysphagia, motor dysfunction, and autonomic failure may simply progress concurrently, representing correlation rather than causation.

### Association between DSS Scores and CSF 5‐HIAA Levels

Serotonin regulates autonomic and motor functions, gastrointestinal motility, and swallowing reflexes,^27^, ^36^, ^37^, ^38^ supporting the hypothesis that serotonin pathway dysregulation could contribute to dysphagia progression in MSA. Previous studies have linked serotonin to motor dysfunction, especially in truncal control, which aligns with neuromotor deficits seen in MSA.^28^ Additionally, serotonin is critical for swallowing‐breathing coordination.^39^, ^40^, ^41^, ^42^ This implies that serotonergic dysfunction may affect both dysphagia and motor‐autonomic failures in MSA, suggesting shared serotonin‐related pathological pathways. Given the association between DSS scores and CSF 5‐HIAA levels, further research into serotonin metabolism biomarkers could shed light on MSA mechanisms and lead to therapeutic strategies targeting the serotonin system, potentially alleviating dysphagia and other symptoms. In this study, the positive correlation between DSS scores and CSF 5‐HIAA levels suggests that serotonin metabolism may influence dysphagia severity in MSA. However, ordinal logistic regression analysis with DSS as the dependent variable identified disease duration and UMSARS Part 4 subscores as the primary determinants of DSS, while CSF 5‐HIAA may serve as an auxiliary associated factor.

### Comparison between the MSA‐P and MSA‐C Groups

In MSA‐P, degeneration of the striatum and substantia nigra leads to parkinsonian symptoms such as rigidity and bradykinesia, which impair swallowing coordination. These dysfunctions result in delayed swallow reflexes, restricted tongue movement, prolonged swallow initiation, and vocal cord dysfunction, thereby increasing the risk of aspiration. In contrast, in MSA‐C, degeneration of the cerebellar and pontine nucleus primarily cause ataxia and disrupt precise muscle control for swallowing. This dysfunction leads to impaired timing of the swallowing process and incomplete food transfer, further increasing the risk of aspiration and dysphagia. The findings of the present study suggest that the DSS scores of MSA‐C patients are correlated with a broader range of clinical indices compared with MSA‐P. This observation may be influenced by differences in both the number of cases and the rate of disease progression. Additionally, the complex interplay of disease‐specific motor symptoms in MSA‐P may further complicate the interpretation of the direct correlation analyses.

### Limitations

This study has several limitations. First, the sample size was relatively small, particularly in the longitudinal analysis. This may affect the generalizability of the findings, especially in terms of detecting subtle longitudinal changes in dysphagia progression. Further studies with larger cohorts are necessary to confirm our results. Second, the variability in disease progression among individual patients may have affected the correlation strength in the longitudinal analyses. Although we applied a log transformation to enhance the sensitivity to subtle changes, individual differences in MSA progression could introduce variability. More frequent and standardized assessment intervals may help clarify these relationships. Third, DSS relies primarily on clinical evaluations without video‐based methods, which may limit its sensitivity in detecting subclinical dysphagia compared with more detailed instrumental assessments, such as VF or VE. Moreover, in our retrospective design, often only a single speech‐language pathologist assessed DSS scores and recorded them in each patient's chart, which could limit the generalizability of our findings. While DSS can be rated by multiple evaluators—particularly during VFSS or endoscopic examinations—our data set did not always confirm whether multiple raters were involved for each case. Furthermore, we acknowledge that the absence of an independent assessment of inter‐rater reliability is a limitation. Although DSS reliability has been favorably assessed by evaluators,^43^, ^44^ our study does not provide new data on this aspect. Future research should consider combining the DSS with these imaging tools to verify the accuracy of the scale in detecting early signs of dysphagia. Finally, the study was conducted at a single institution, which may have introduced a selection bias related to regional or institutional diagnostic and treatment practices. Multi‐center studies could mitigate this limitation by providing a more representative sample of patients with MSA.

In summary, the simplicity and ease of use of the DSS in routine clinical practice make it suitable for regular patient monitoring. It allows clinicians to track changes in swallowing function without specialized equipment. Integrating DSS assessments into routine care pathways for MSA patients could facilitate the early identification of swallowing dysfunction, enabling timely interventions such as dietary modifications and preventive strategies against aspiration. Given its sensitivity and specificity, the DSS could be valuable for disease management and as a functional outcome measure in clinical trials examining MSA progression.

## Author Roles

(1) Research project: A. Conception, B. Organization, C. Execution; (2) Statistical Analysis: A. Design, B. Execution, C. Review and Critique; (3) Manuscript: A. Writing of the first draft, B. Review and Critique.

R.N.: 1A, 1B, 1C, 2A, 2B, 3A.

Y.M.: 1A, 1B, 1C, 2C.

K.K.: 1A, 1B, 1C, 2C.

J.Y.: 2C.

Y.I.: 3B.

S.S.: 3B.

M.I.: 1A, 1B, 1C.

Y.O.: 3B.

H.W.: 1A, 1B, 1C, 2C, 3A, 3B.

## Disclosures

**Ethical Compliance Statement:** We confirm that we have read the Journal's position on issues involved in ethical publication and affirm that this work is consistent with those guidelines. This study was approved by the Ethics Committee of Fujita Health University Hospital (HM23‐296). Written informed consent, including the provision for opt‐out, was obtained from all participants prior to their inclusion. This study conformed to the principles described in the Declaration of Helsinki.

**Funding Sources and Conflict of Interest:** The authors declare that there are no funding sources or conflicts of interest relevant to this work.

**Financial Disclosures for the Previous 12 Months:** R.N. and M.I. have no financial conflicts of interest to disclose. Y.M. has received research support from Nihon Medi‐Physics Co., Ltd. via the researcher‐initiated clinical research support program. K.K. reports a grant from the Japan Society for the Promotion of Science (JSPS) Overseas Research Fellowship. J.Y. has received research funding from Honda R&D Co., Ltd, AMED (Grant Numbers: JP21wm0425008, JP21wm0425017, JP21wm0425019, and JP23tm0524001), and JSPS KAKENHI (Grant Numbers: JP23K20771 and JP24K15184). I.Y. has received grants from the JSPS (KAKENHI 23K10414, 22K11462, and 24K02736). S.S. reports Japanese Patent No. 7495063 and a grant from the JSPS (KAKENHI 22K11407). Y.O. has received research funding from MATSUNAGA MANUFACTORY Co., Ltd., Chuo Spring Co., Ltd., Tokyo Metropolitan Institute of Medical Science, Mitsubishi Electric Corporation, Unicharm Corporation, Flicfit inc., PLATZ Co., Ltd., TAK Co., Ltd., NTT DOCOMO, INC., Toyota Motor Corporation, Toyota Tsusho Corporation, Magic Shields, Inc., SINTOKOGIO, LTD., FRONTEO, Inc., and Bando Chemical Industries, Ltd., and has also received the following research grants: Grants‐in‐Aid for Scientific Research, Health Labour Sciences Research Grant, Knowledge Hub Aichi Priority Research Project (4th term), Application Promotion Project of Care and Rehabilitation Robot, The Platform project of Development, Trial and Promotion of Nursing Care Robots, New Energy and Industrial Technology Development Organization, Aichi Digital Health Project, and Bioelectromagnetic environment research and evaluation technology research on radio wave safety. H.W. received lecture fees from Kyowa Kirin, Takeda Pharmaceutical, Sumitomo Pharma, Eisai, Abbvie, and Ono pharmaceutical.

## Supporting information


**TABLE S1.** Correlations between DSS and clinical indices using Spearman's rank correlation coefficient and FDR correction in MSA‐P.
**TABLE S2.** Correlations between DSS and clinical indices using Spearman's rank correlation coefficient and FDR correction in MSA‐C.

## Data Availability

The data that support the findings of this study are available from the corresponding author upon reasonable request.
