# Supplementary material for: Correlations between Dysphagia Severity Scale Scores and Clinical Indices in Individuals with Multiple System Atrophy
Source: Mov Disord Clin Pract. 2025 Mar 25;12(8):1086–96. doi: 10.1002/mdc3.70055 (PMC12371456; doi:10.1002/mdc3.70055)
Supplement: Supplementary file 1 — TABLE S1. Correlations between DSS and clinical indices using Spearman's rank correlation coefficient and FDR correction in MSA‐P. TABLE S2. Correlations between DSS and clinical indices using Spearman's rank correlation coefficient and FDR correction in MSA‐C. [file MDC3-12-1086-s001.pptx]

## Slide 1
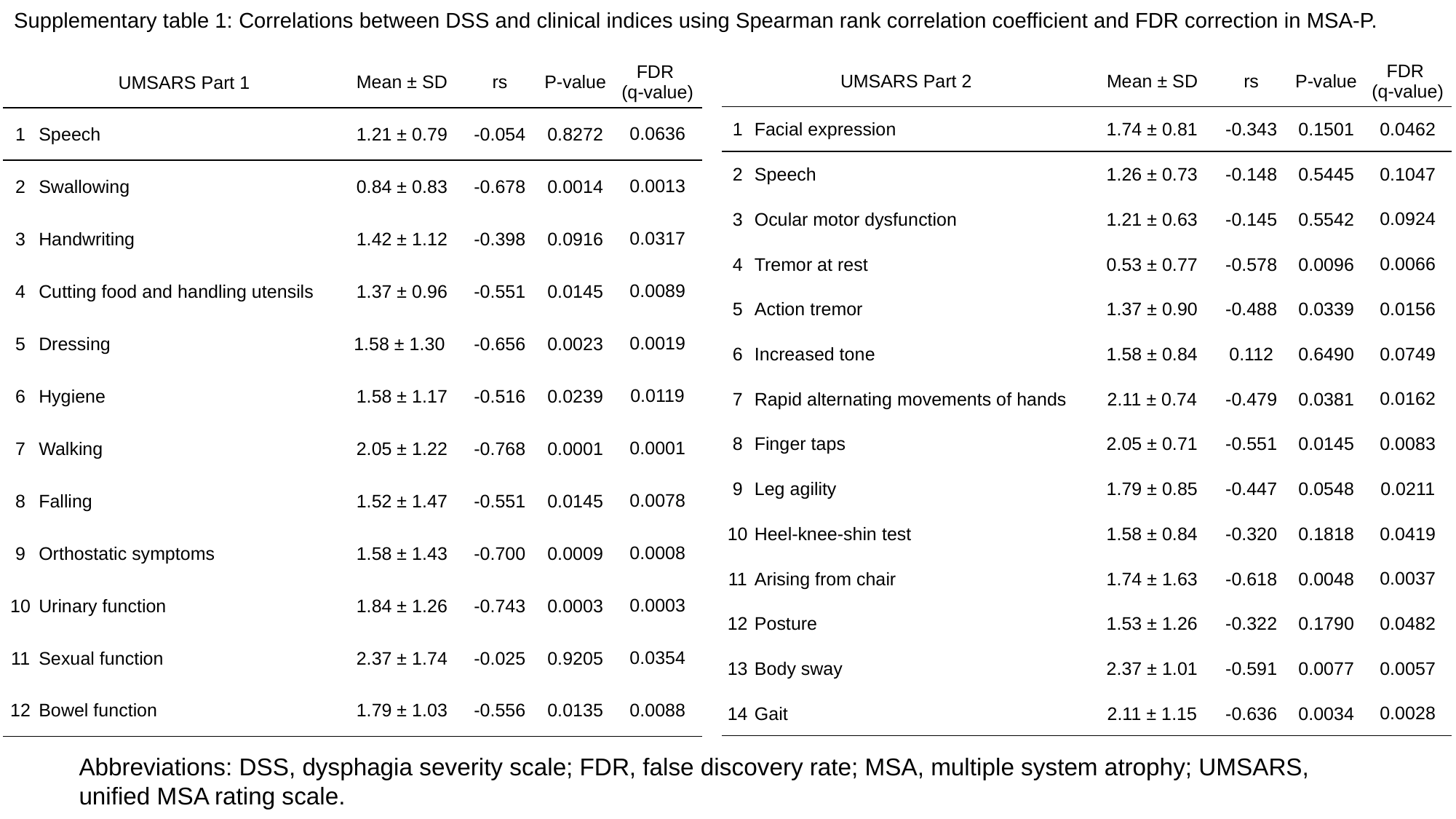

Supplementary table 1: Correlations between DSS and clinical indices using Spearman rank correlation coefficient and FDR correction in MSA-P.
| UMSARS Part 2 | | Mean ± SD | rs | P-value | FDR (q-value) |
| --- | --- | --- | --- | --- | --- |
| 1 | Facial expression | 1.74 ± 0.81 | -0.343 | 0.1501 | 0.0462 |
| 2 | Speech | 1.26 ± 0.73 | -0.148 | 0.5445 | 0.1047 |
| 3 | Ocular motor dysfunction | 1.21 ± 0.63 | -0.145 | 0.5542 | 0.0924 |
| 4 | Tremor at rest | 0.53 ± 0.77 | -0.578 | 0.0096 | 0.0066 |
| 5 | Action tremor | 1.37 ± 0.90 | -0.488 | 0.0339 | 0.0156 |
| 6 | Increased tone | 1.58 ± 0.84 | 0.112 | 0.6490 | 0.0749 |
| 7 | Rapid alternating movements of hands | 2.11 ± 0.74 | -0.479 | 0.0381 | 0.0162 |
| 8 | Finger taps | 2.05 ± 0.71 | -0.551 | 0.0145 | 0.0083 |
| 9 | Leg agility | 1.79 ± 0.85 | -0.447 | 0.0548 | 0.0211 |
| 10 | Heel-knee-shin test | 1.58 ± 0.84 | -0.320 | 0.1818 | 0.0419 |
| 11 | Arising from chair | 1.74 ± 1.63 | -0.618 | 0.0048 | 0.0037 |
| 12 | Posture | 1.53 ± 1.26 | -0.322 | 0.1790 | 0.0482 |
| 13 | Body sway | 2.37 ± 1.01 | -0.591 | 0.0077 | 0.0057 |
| 14 | Gait | 2.11 ± 1.15 | -0.636 | 0.0034 | 0.0028 |
| UMSARS Part 1 | | Mean ± SD | rs | P-value | FDR (q-value) |
| --- | --- | --- | --- | --- | --- |
| 1 | Speech | 1.21 ± 0.79 | -0.054 | 0.8272 | 0.0636 |
| 2 | Swallowing | 0.84 ± 0.83 | -0.678 | 0.0014 | 0.0013 |
| 3 | Handwriting | 1.42 ± 1.12 | -0.398 | 0.0916 | 0.0317 |
| 4 | Cutting food and handling utensils | 1.37 ± 0.96 | -0.551 | 0.0145 | 0.0089 |
| 5 | Dressing | 1.58 ± 1.30 | -0.656 | 0.0023 | 0.0019 |
| 6 | Hygiene | 1.58 ± 1.17 | -0.516 | 0.0239 | 0.0119 |
| 7 | Walking | 2.05 ± 1.22 | -0.768 | 0.0001 | 0.0001 |
| 8 | Falling | 1.52 ± 1.47 | -0.551 | 0.0145 | 0.0078 |
| 9 | Orthostatic symptoms | 1.58 ± 1.43 | -0.700 | 0.0009 | 0.0008 |
| 10 | Urinary function | 1.84 ± 1.26 | -0.743 | 0.0003 | 0.0003 |
| 11 | Sexual function | 2.37 ± 1.74 | -0.025 | 0.9205 | 0.0354 |
| 12 | Bowel function | 1.79 ± 1.03 | -0.556 | 0.0135 | 0.0088 |
Abbreviations: DSS, dysphagia severity scale; FDR, false discovery rate; MSA, multiple system atrophy; UMSARS, unified MSA rating scale.

## Slide 2
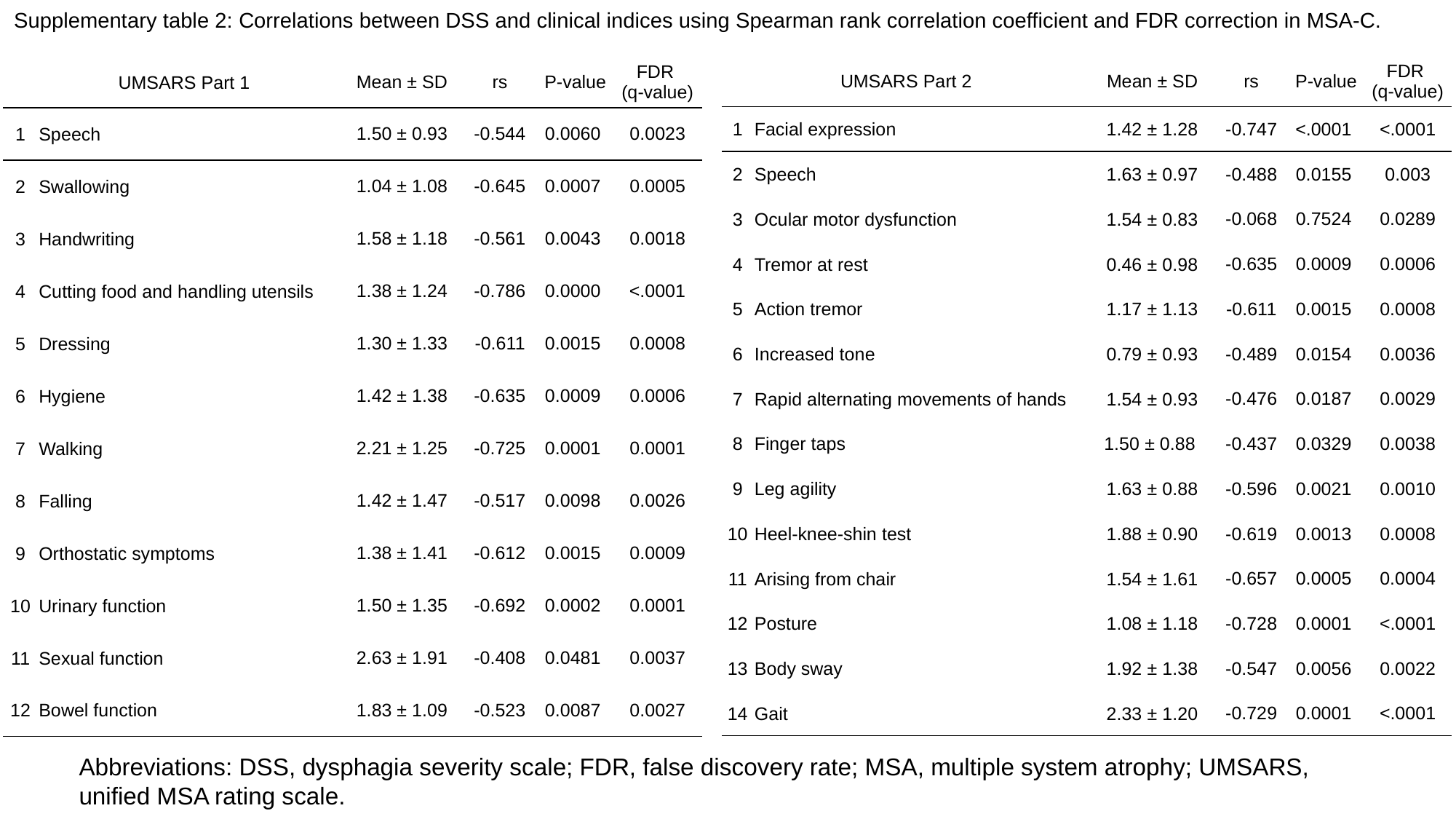

Supplementary table 2: Correlations between DSS and clinical indices using Spearman rank correlation coefficient and FDR correction in MSA-C.
| UMSARS Part 2 | | Mean ± SD | rs | P-value | FDR (q-value) |
| --- | --- | --- | --- | --- | --- |
| 1 | Facial expression | 1.42 ± 1.28 | -0.747 | <.0001 | <.0001 |
| 2 | Speech | 1.63 ± 0.97 | -0.488 | 0.0155 | 0.003 |
| 3 | Ocular motor dysfunction | 1.54 ± 0.83 | -0.068 | 0.7524 | 0.0289 |
| 4 | Tremor at rest | 0.46 ± 0.98 | -0.635 | 0.0009 | 0.0006 |
| 5 | Action tremor | 1.17 ± 1.13 | -0.611 | 0.0015 | 0.0008 |
| 6 | Increased tone | 0.79 ± 0.93 | -0.489 | 0.0154 | 0.0036 |
| 7 | Rapid alternating movements of hands | 1.54 ± 0.93 | -0.476 | 0.0187 | 0.0029 |
| 8 | Finger taps | 1.50 ± 0.88 | -0.437 | 0.0329 | 0.0038 |
| 9 | Leg agility | 1.63 ± 0.88 | -0.596 | 0.0021 | 0.0010 |
| 10 | Heel-knee-shin test | 1.88 ± 0.90 | -0.619 | 0.0013 | 0.0008 |
| 11 | Arising from chair | 1.54 ± 1.61 | -0.657 | 0.0005 | 0.0004 |
| 12 | Posture | 1.08 ± 1.18 | -0.728 | 0.0001 | <.0001 |
| 13 | Body sway | 1.92 ± 1.38 | -0.547 | 0.0056 | 0.0022 |
| 14 | Gait | 2.33 ± 1.20 | -0.729 | 0.0001 | <.0001 |
| UMSARS Part 1 | | Mean ± SD | rs | P-value | FDR (q-value) |
| --- | --- | --- | --- | --- | --- |
| 1 | Speech | 1.50 ± 0.93 | -0.544 | 0.0060 | 0.0023 |
| 2 | Swallowing | 1.04 ± 1.08 | -0.645 | 0.0007 | 0.0005 |
| 3 | Handwriting | 1.58 ± 1.18 | -0.561 | 0.0043 | 0.0018 |
| 4 | Cutting food and handling utensils | 1.38 ± 1.24 | -0.786 | 0.0000 | <.0001 |
| 5 | Dressing | 1.30 ± 1.33 | -0.611 | 0.0015 | 0.0008 |
| 6 | Hygiene | 1.42 ± 1.38 | -0.635 | 0.0009 | 0.0006 |
| 7 | Walking | 2.21 ± 1.25 | -0.725 | 0.0001 | 0.0001 |
| 8 | Falling | 1.42 ± 1.47 | -0.517 | 0.0098 | 0.0026 |
| 9 | Orthostatic symptoms | 1.38 ± 1.41 | -0.612 | 0.0015 | 0.0009 |
| 10 | Urinary function | 1.50 ± 1.35 | -0.692 | 0.0002 | 0.0001 |
| 11 | Sexual function | 2.63 ± 1.91 | -0.408 | 0.0481 | 0.0037 |
| 12 | Bowel function | 1.83 ± 1.09 | -0.523 | 0.0087 | 0.0027 |
Abbreviations: DSS, dysphagia severity scale; FDR, false discovery rate; MSA, multiple system atrophy; UMSARS, unified MSA rating scale.
